# Hydrolysis capacity of different sized granules in a full-scale aerobic granular sludge (AGS) reactor

**DOI:** 10.1016/j.wroa.2022.100151

**Published:** 2022-07-31

**Authors:** Sara Toja Ortega, Lenno van den Berg, Mario Pronk, Merle K. de Kreuk

**Affiliations:** a Section Sanitary Engineering, Department of Water Management, Delft University of Technology, Stevinweg 1, Delft 2628CN, the Netherlands; b Department of Biotechnology, Delft University of Technology, Van der Maasweg 9, Delft, HZ 2629, the Netherlands; c Royal HaskoningDHV, Laan 1914 35, Amersfoort, AL 3800, the Netherlands

**Keywords:** Wastewater treatment, Aerobic granular sludge, Hydrolysis, Polymeric substrates, Activity staining, Biomass segregation, AGS, aerobic granular sludge, AS, activated sludge, COD, chemical oxygen demand, EBPR, enhanced biological phosphorus removal, EPS, extracellular polymeric substances, FISH, fluorescence in situ hybridization, GAO, glycogen-accumulating organism, PAO, polyphosphate-accumulating organism, SBR, sequencing batch reactor, SND, simultaneous nitrification-denitrification, SRT, solids retention time, TSS, total suspended solids, VFA, volatile fatty acid, VSS, volatile suspended solids, WWTP, wastewater treatment plant

## Abstract

In aerobic granular sludge (AGS) reactors, granules of different sizes coexist in a single reactor. Their differences in settling behaviour cause stratification in the settled granule bed. In combination with substrate concentration gradients over the reactor height during the anaerobic plug-flow feeding regime, this can result in functional differences between granule sizes. In this study, we compared the hydrolytic activity in granules of 4 size ranges (between 0.5 and 4.8 mm diameter) collected from a full-scale AGS installation. Protease and amylase activities were quantified through fluorescent activity assays. To visualise where the hydrolytic active zones were located within the granules, the hydrolysis sites were visualized microscopically after incubating intact and sliced granules with fluorescent casein and starch. The microbial community was studied using fluorescent *in situ* hybridization (FISH) and sequencing. The results of these assays indicated that hydrolytic capacity was present throughout the granules, but the hydrolysis of bulk substrates was restricted to the outer 100 µm, approximately. Many of the microorganisms studied by FISH, such as polyphosphate and glycogen accumulating organisms (PAO and GAO), were abundant in the vicinity of the hydrolytically active sites. The biomass-specific hydrolysis rate depended mainly on the available granule surface area, suggesting that different sized granules are not differentiated in terms of hydrolytic capacity. Thus, the substrate concentration gradients that are present during the anaerobic feeding in AGS reactors do not seem to affect hydrolytic activity at the granule surfaces. In this paper, we discuss the possible reasons for this and reflect about the implications for AGS technology.

## Highlights

• Aerobic granules of different sizes exhibit similar surface-specific hydrolysis.
• Aerobic granules of different sizes differ in their microbial community composition.
• Activity staining and FISH are combined to characterize hydrolysis sites.
• Hydrolysis of influent polymers mainly occurs in the outer layer of aerobic granules (<100 µm depth).
• PAOs and GAOs are located nearby hydrolysis sites.

## Graphical abstract

**Figure:**
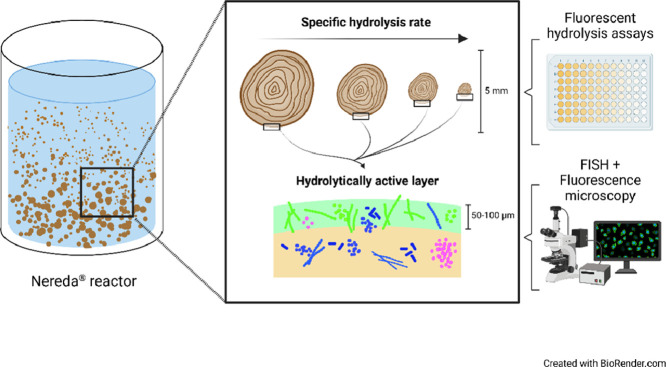

## 1 Introduction

Aerobic granular sludge (AGS) is an advanced wastewater treatment technology, which uses granular biofilms for removing pollutants from the wastewater. Besides removing COD, AGS can perform enhanced biological phosphorus removal (EBPR) and biological nitrogen removal (Bengtsson et al., 2018; de Kreuk et al., 2005; Pronk et al., 2015b). In most full-scale applications, the treatment involves anaerobic uptake of COD followed by aerobic/anoxic biomass growth. This is achieved by applying a sequencing batch reactor (SBR) cycle that consists of three phases: anaerobic feeding, reaction and settling (Bengtsson et al., 2018; Derlon et al., 2016; Pronk et al., 2015b). At the end of the settling phase, slow settling sludge is selectively discharged (referred to as selection spill). The treated water is discharged during the next feeding phase, displaced by the influent volume fed from the reactor bottom. Plug-flow feeding is important to avoid mixing of the untreated influent and treated effluent, and to provide high local substrate concentrations (de Kreuk and van Loosdrecht, 2004; Pronk et al., 2015a).

The employed SBR cycle, besides ensuring adequate wastewater treatment, influences biomass differentiation. AGS reactors are usually composed of granules of different sizes and flocs. In AGS literature, a range of granule diameters has been reported, varying from 0.2 mm to more than 6 mm (Coma et al., 2012; Lanham et al., 2012; Layer et al., 2019; Stes et al., 2021; Toja Ortega et al., 2021a). The selection spill wastes mainly flocs and small granules, which creates biomass fractions with different SRTs in the same reactor (Ali et al., 2019; Guo et al., 2020). Large granules can have an SRT of months, while on the opposite end flocculent sludge is retained for only a few days (Ali et al., 2019). In addition, plug-flow feeding results in higher substrate concentrations at the bottom of the sludge bed. Larger granules, with a higher settling velocity, are more likely to end up in the bottom of the settled bed during feeding, experiencing higher substrate concentrations than small granules (van Dijk et al., 2022). These conditions could result in functional differences between the granules of different sizes, coupled to their differences in mass-transfer resistance. For instance, in the full-scale AGS plant of Garmerwolde, the Netherlands, polyphosphate-accumulating organisms (PAO) and glycogen-accumulating organisms (GAO) were more concentrated in the larger granules (Ali et al., 2019). Such selection was attributed to the long retention of large granules and their preferential uptake of volatile fatty acids (VFA) during feeding. Moreover, larger granule sizes are more suitable for simultaneous nitrification-denitrification (SND) because of their larger anoxic volume during the reaction phase (Layer et al., 2020). In smaller granules, on the other hand, nitrifying organisms are enriched due to a larger relative aerobic volume (Nguyen Quoc et al., 2021). Similarly, granule size could be important for the hydrolysis of slowly biodegradable substrates. However, biomass differentiation regarding hydrolysis has not been studied in detail yet.

Hydrolysis is a key process to consider when studying the fate of polymeric substrates during wastewater treatment. These substrates make up the largest COD fraction in municipal wastewater, and must be extracellularly hydrolysed to compounds of smaller molecular weight (<1 kDa) to be assimilated by microorganisms (Ferenci, 1980; Hollibaugh and Azam, 1983). Previous experimental work found hydrolysis to be mainly biomass-bound, rather than occurring in the reactor bulk liquid (Confer and Logan, 1998; Goel et al., 1998; Janning et al., 1998, 1997; Mosquera-Corral et al., 2003). In full-scale AGS, aerobic granules showed significant hydrolytic activity, suggesting that wastewater polymers can be utilized by granules (Toja Ortega et al., 2021a). However, the limited diffusion of polymers into the granules affects their hydrolysis, resulting in lower biomass-specific hydrolysis rates in granules compared to flocculent sludge. Moreover, several studies suggest that most of the particulates are captured by flocculent sludge, and the granules are only accessible for polymeric substrates of smaller size (i.e. colloidal and soluble) (Layer et al., 2020; Ranzinger et al., 2020). Those polymeric molecules can be present in the wastewater or originate from the conversion of the particulate COD. Thus, it is still uncertain how relevant granules are for hydrolysing influent polymers and which granule volume is hydrolytically active. Those points should be addressed to clarify the contribution of polymeric COD to nutrient removal processes and stable granule growth.

In addition, it is not known whether all granules within an AGS reactor have a similar hydrolysis capacity. Smaller granules would presumably hydrolyse influent substrates at a higher rate due to their higher available surface area (Toh et al., 2003). Nevertheless, the hydrolytic activity of granules of different sizes might be influenced by factors other than mass transfer of the substrate. For instance, the abovementioned SRT differences and substrate concentration gradients in the sludge bed during feeding could support the enrichment of hydrolysing organisms and enzymes in some specific granule sizes. Exploring differences in hydrolytic activity along the AGS bed would add to the current knowledge of biomass differentiation in AGS reactors. Furthermore, the distribution of hydrolytic activity may reflect the removal mechanism of particulate substrates in AGS. Understanding the differences in microbial activity on different granule size ranges can ultimately aid process control.

In this study we explored the location of hydrolysis in AGS on granule level. First, we studied whether granules of different sizes within an AGS bed are differentiated in terms of their hydrolytic potential. We compared the biomass-specific hydrolysis rate of granules of different sizes, focussing on protease and amylase activities. These enzyme groups were targeted based on previous studies that showed significant protease and α-glucosidase activities on AGS (Toja Ortega et al., 2021a, 2021b). In addition, we investigated the location of hydrolysis within granules, to gain insight into substrate penetration depth and the distribution of enzymes throughout the granules. Microbial groups of interest were identified via fluorescence *in situ* hybridization (FISH) to study their localization relative to the hydrolysis sites. The hydrolytic activity results from each granule size fraction were finally evaluated in relation to other granule characteristics, such as granule density and settling velocity, substrate uptake rate, and microbial community composition. Based on these observations, we reflect on the implications of our findings for particulate hydrolysis in AGS and full-scale operation.

## 2 Materials & methods

### 2.1 Sludge sampling and processing

Aerobic granules were harvested in wastewater treatment plant (WWTP) Utrecht, the Netherlands. The plant treats domestic wastewater in 6 Nereda® reactors of 12,000 m^3^, designed by Royal HaskoningDHV and operated by the Dutch district water authority Hoogheemraadschap de Stichtse Rijnlanden. The average flow during the sampling period was 74,700 m^−3^ d^−1^, and the reactors are operated with a sludge loading of 0.05 kg COD kg VSS^−1^ d^−1^. The full-scale reactors were sampled after at least 20 min of aeration to ensure a homogeneous mixed liquor sample.

The granules were separated into four size fractions by sieving the reactor mixed liquor through different mesh sizes. The resulting granule size fractions consisted of the following diameter ranges: 0.5−1 mm, 1.6−2 mm, 2.5−3.15 mm, and 4−4.8 mm. Non-overlapping fractions were used in the study to facilitate the comparison between granules of different size. The granule fractions derived from sieving were cleaned based on settling to remove non-granule material, like husk and fibers. To do so, granules were transferred to a beaker and tap water was added. The content of the beaker was mixed by stirring. After a few seconds of settling time, granules were at the bottom of the beaker and the liquid only contained the non-granular fraction that was subsequently discarded. The process was repeated for 5−10 times until the discarded liquid was clean. The resulting granule size fractions were stored at 4°C until use, for a maximum of 2 days. Before the assays, the granules were acclimatized for half an hour in 10 mM Tris-HCl buffer (pH 7.8) at room temperature (20°C).

### 2.2 Protease and amylase assays

Protease and amylase activities of the granules were quantified using fluorogenic substrates. These substrates enable studying the activity on a single granule scale, in a 96-well-plate reader. Furthermore, studying the hydrolysis of real wastewater polymeric substrates is extremely challenging due to their complexity and variability. Therefore, surrogates were used to provide a reproducible experimental setup and study the hydrolytic capacity of granules in detail. The hydrolytic activity assays were based on the protein hydrolysis assay described by Van Gaelen et al. (2020). Protease activity was measured using the BODIPY® FL casein substrate (EnzChek Protease Assay Kit E6638, Thermofisher scientific, Waltham, Massachusetts, USA). Amylase activity was measured using the BODIPY® FL DQ™ starch substrate (EnzChek™ Ultra Amylase Assay Kit E33651, Thermofisher scientific, Waltham, Massachusetts, USA). The substrates were prepared following kit instructions, rendering a protease solution of 10 mg BODIPY® casein/L and an amylase substrate of 200 mg BODIPY® starch/L. There was a modification: amylase substrate was prepared using the digestion buffer provided in the protease assay (10 mM Tris-HCl, pH 7.8, 0.1 mM sodium azide), in order to keep the same assay conditions. The digestion buffer used in the assay was also the 10 mM Tris-HCl provided in the protease assay.

Two types of sample were studied per granule size fraction: intact granules and crushed granules. The latter were used to determine the hydrolytic capacity of the sludge minimizing mass transfer limitation. Each granule size fraction was studied on a separate run, using a 96-well plate. In each run, hydrolysis rates were monitored in 4 wells containing crushed sludge, as well as in 20 wells containing one intact granule per well. For the intact granule samples, 100 µl of digestion buffer and one granule were added to the assay wells. The crushed granules were prepared by using a Potter-Elvehjem tissue grinder, and diluted to an approximate concentration of 1 g TSS/L using digestion buffer. Then, 100 µl of crushed sludge sample were added to the assay wells. To start the assay, 50 µl of substrate was added to all the samples. Besides the samples, controls were added in each assay. Blanks were prepared for each type of sludge, by adding digestion buffer instead of substrate. Standard samples were also included for each type of sludge, by adding a known concentration of hydrolysed substrate (standard) instead of fresh substrate. The standards were prepared by hydrolysing an aliquot of the substrate solution with commercial enzyme (P5985 and 10065, Sigma-Aldrich, St. Louis, MO, USA).

Hydrolytic activity was monitored in a 96-well plate reader (FLUOstar Galaxy Multi-functional Microplate Reader, BMG LABTECH, Ortenberg, Germany). The reader measures the fluorescence intensity in each of the 96 wells in defined time intervals. Each experiment ran for approximately 1.5 h, with fluorescence readings every 5 min. For both substrates, the Ex/Em wavelengths 485/538 nm were used. The experiments were performed with acclimatised granules at room temperature, with linear mixing. Per hydrolytic activity assay, all the granules were fractionated from the same mixed liquor sample batch and their activities were measured on the same day.

### 2.3 Calculation of hydrolysis rates

The fluorescence intensities from the individual wells were analysed through linear regression. The fluorescence intensities of each well were normalized with the fluorescence intensity of the standards. The linear range of the time-resolved fluorescence curve was visually selected to calculate the hydrolysis rate, expressed as fluorescence (arbitrary units) per hour. The activity per g VSS was calculated by estimating the amount of biomass in each well. In the crushed sludge samples, the VSS concentration of the samples was directly measured following Standard Methods (APHA, 2005). For the granule samples, the amount of biomass per well was variable, and therefore the VSS per well was estimated, instead of measured directly. The estimation was done as follows. First, granule volume was estimated through light microscopy. The plate was placed under a digital microscope (VHX-700F, Keyence, Mechelen, Belgium) and an image of each granule was captured. The images were analysed using the microscope software, to extract the circle equivalent diameter of the granules. The granule volume was then calculated from this diameter, assuming spherical granules. Finally, the granule volume was translated to VSS using granule biomass density, determined separately (see Materials & Methods, granule density measurements). Hydrolytic activity was also expressed relative to granule surface area. The surface area, too, was calculated based on the circle equivalent diameter determined via image analysis of each granule separately. The obtained biomass-relative and surface-relative rates were normalized against the highest measured rate to simplify visualizations, given that the fluorescence units were by definition arbitrary.

### 2.4 Enzyme staining and microscopic examination

#### 2.4.1 Identification of hydrolytically active sites

BODIPY® FL casein and BODIPY® FL DQ™ starch were also used for localizing hydrolytically active sites in the granules. The objective of these experiments was to assess the location within the granule at which polymers from the bulk can be hydrolysed. The procedure was based on the enzyme staining method described for activated sludge (Xia et al., 2008b, 2008a, 2007). We modified the protocol to stain the biofilm instead of flocculent sludge. Substrate solutions of 100 mg/L were prepared diluting the substrates in Tris-HCl buffer (10 mM, pH 7.8). The substrate concentration and incubation time were chosen based on preliminary assays (Supplementary information, Table S1). Granules were incubated with fluorescent substrates in 2 mL sample tubes (Eppendorf, Hamburg, Germany). Approximately 120 mg of fresh granules (5 mg TSS) were added to 500 µl of digestion buffer and 250 µl of substrate solution. That rendered a substrate to sludge ratio of 5 µg substrate per g TSS. Samples were incubated for two hours, in the dark and with orbital shaking at 120 rpm (Fisherbrand Seastar, Thermo Fisher Scientific, Waltham, USA).

After incubation with fluorescent substrates, the granules were transferred to freeze-drying medium (Neg-50^TM^, Thermo Fisher Scientific, Waltham, USA) and stored at 4°C overnight, protected from light. Then, the granules were sectioned in a Leica CM1900 cryostat (Leica microsystems, Nussloch, Germany). The temperature of the blade and the sample holder was -22°C, and the granules were sectioned in 20 µm-thick coupes. Granule sections were spread on poly-lysine coated slides and air-dried in the dark. Finally, the sections were examined using a Zeiss Axioplan 2 epifluorescence microscope (Zeiss, Oberkochen, Germany). The fluorescent signals from the hydrolysed BODIPY® FL casein and BODIPY® FL DQ™ starch were visualized and captured using a FLUOS filter (excitation 485 nm, emission 515-565 nm).

#### 2.4.2 Distribution of hydrolytic enzymes throughout the granule

A variation of the above described method was used to explore the distribution of enzymes throughout the granule. Fresh granules were sliced using a scalpel, mounted in a microscope slide, and incubated with the fluorogenic substrates. The granule sections were covered with 10 µl of a 2:1 buffer:substrate solution and incubated in the dark for 2 h. Then, the substrate solution was replaced by digestion buffer and the slides were observed on the fluorescence microscope.

### 2.5 Fluorescent *in situ* hybridization (FISH)

After inspecting the activity staining, the granule sections were fixed in ethanol (50%) for 2 h. Ethanol fixing was used independently of the microbial group targeted by FISH (Gram negative or Gram positive), as described in Xia et al. (2007).

The following probes were used: EUBmix, staining most bacteria (a mixture of EUB338, EUB338-II and EUB338-III) (Amann et al., 1990; Daims et al., 1999); PAOmix, targeting *Ca. Accumulibacter*-related polyphosphate-accumulating organisms (PAO) (a mixture of PAO462, PAO651, and PAO846) (Crocetti et al., 2000); GAOmix, targetting glycogen-accumulating organisms (GAO) (a mixture of GAOQ431 and GAOQ989 probes) (Crocetti et al., 2002); Actino-221, targeting actinobacterial PAO related to genus *Tetrasphaera* (Kong et al., 2005); CFX1223 (Björnsson et al., 2002) and GNSB-941 (Gich et al., 2001), targeting most members of the phylum *Chloroflexi*; and Bac111 targeting activated sludge clones of the family *Saprospiraceae* (Kong et al., 2007). Detailed information of all these probes is given in probeBase (Loy et al., 2003). All the probes were Cy3 or Cy5 labelled.

FISH staining was performed as described in Bassin et al. (2011). Samples were incubated for 2 h with the FISH probes. The stained slides were mounted with VECTASHIELD® antifade mounting medium (Vector laboratories, Burlingame, CA, USA), and inspected on the epifluorescence microscope. The hybridised cells were visualized using the filters Cy3 (excitation 546 nm, emission 575−640 nm) and Cy5 (excitation 575−625 nm, emission 660−710 nm).

### 2.6 Microbial diversity analysis

Three sludge samples were collected at different points of each of the studied reactors (R1, R3 and R4). Each of the triplicate samples was sieved separately using the same procedure as described in section 2.1. 15 mL of the sieved granules (roughly 300 mg TSS) of each size fraction were homogenized using a tissue grinder. The homogenized samples were centrifuged at 14,000 g for 5 min and approximately 2 mg TSS of the resulting pellet were added to the extraction tubes. DNA was then extracted as described in Toja Ortega et al. (2021b).

The V3-V4 regions of the 16S rRNA gene were amplified and paired-end sequenced in an llumina NovaSeq 6000 platform by Novogene (Beijing, China). The primer set 341F [5′– CCTAYGGGRBGCASCAG–3′] and 806R [5′– GGACTACNNGGGTATCTAAT–3′] was used. The raw reads were deposited in the National Center for Biotechnology Information (NCBI) Sequence Read Archive (SRA) on BioProject PRJNA837281.

The trimmed and merged sequences provided by Novogene were processed using QIIME2, version 2020.2 (Bolyen et al., 2019). The sequences were quality-filtered using Deblur (Amir et al., 2017), trimming the sequences the 3′ end at position 237 (parameter p-trim-length). With the remaining sequences, alpha and beta diversity metrics were generated, and differences in diversity between granule sizes were assessed using PERMANOVA (Anderson, 2001). A p-value of 0.05 was used as cut-off for significance. Finally, taxonomic affiliation of the sequences was determined by aligning the sequences to the MiDAS 3.6 database (Nierychlo et al., 2020). Sample subsetting, visualization and further statistical analysis was performed in R, using the Phyloseq and Ampvis2 packages (Andersen et al., 2018; McMurdie and Holmes, 2013). Differences in the relative abundance of the 20 most abundant genera were studied performing a Wald test. The relative abundances in the smallest and largest granules were compared. Differences were considered significant with a p value below the Bonferroni-corrected *p* value of 0.05 (*p* = 0.05/number of comparisons).

### 2.7 Additional characterization of the granule fractions

#### 2.7.1 PAO activity

The activity of PAO was assessed in the four granule size fractions through batch tests. The assay was performed in triplicate. The granules, which had been stored anaerobically overnight, were acclimated by aerating for 35 min. Then, they were crushed using a Potter-Elvehjem tissue grinder and buffered with 20 mM Tris-HCl (pH 7.5). The assays were conducted in 500 mL bottles, with a final sludge concentration of approximately 3 g VSS/L. Sodium acetate was added to a final concentration of 170 mg COD/L. The bottles were sealed with rubber stoppers and flushed with N_2_ for two minutes to provide anaerobic conditions. The samples were incubated for two hours in a Fisherbrand Seastar orbital shaker at 120 rpm, taking samples regularly (every 6−10 min). Samples were filtered using 0.45 µm PES syringe filters. The acetate concentration of the samples was measured in a gas chromatographer equipped with an FID detector (7890A GC; Agilent Technologies, Santa Clara, CA, USA). Phosphate concentration was determined colorimetrically using the USEPA method 365.1, in an AQ400 discrete analyser (Seal analytical, Mequon, WI, USA). The TSS and VSS concentration of the sludge solutions used in the assay were determined following standard methods (APHA, 2005).

#### 2.7.2 Settling tests

The terminal settling velocity of granules was studied in a measuring cylinder with an internal diameter of 7.94 cm and a height of 42.1 cm, filled with tap water. Granules were placed individually at the top of the cylinder and their settling was recorded using a camera (GoPro Hero Session 4, San Mateo, CA, USA). Around 50 granules were examined per size fraction.

#### 2.7.3 Granule biomass density measurements

Granule biomass density was measured using the modified dextran blue method, as described in van den Berg et al. (2022). The density measurements were performed in 200 mL samples, and each granule size fraction was measured in triplicate.

## 3 Results and discussion

### 3.1 Granule size effect on hydrolysis rate

**Fig. 1:**
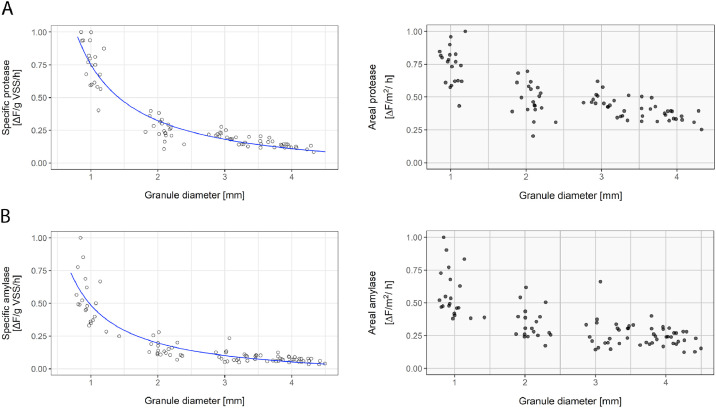
**Hydrolysis rates measured in granules between 0.5 and 4.8 mm.** (a) Protease activity as a function of granule diameter; and (b) amylase activity as a function of granule diameter. In the left graphs, biomass specific activity is shown, as increase in fluorescence per gram VSS per hour. In blue, the inverse proportional fit of the data is shown (y = k / x). The right graphs show the surface-specific hydrolytic activity, as increase in fluorescence per granule surface (m^2^) per hour. Each data point represents one granule.

The hydrolysis capacity of different sized aerobic granules was studied using fluorescent protease and amylase assays. Biomass-specific hydrolytic activity (fluorescence [ΔF]/g VSS/h) was inversely proportional to granule diameter (Fig. 1). This relationship can be explained by the changes in surface/volume ratio with respect to granule diameter, (SA/V ∝ d^−1^, for a perfect sphere). The areal hydrolysis rate (ΔF/m^2^/h) somewhat decreased with increasing granule size too (Fig. 1). Hence, the smaller granules had a higher biomass-specific hydrolytic activity, supported mainly by their proportionally higher available surface area and enhanced by a higher activity per surface area. According to these results, the hydrolytic capacity that potentially could degrade influent particulates can be doubled by halving the average granule diameter in an AGS reactor.

The different biomass-specific rates indicated that hydrolysis was, as described previously, conditioned by mass transfer. In WWTP Garmerwolde, crushed granules and flocs showed roughly twice as high hydrolytic activity as intact granules (e.g., protease rates were 0.016 and 0.017 µmol product mg VSS^−1^ h^−1^ on crushed granules and flocs, and 0.007 µmol product mg VSS^−1^ h^−1^ on intact granules) (Toja Ortega et al., 2021a). Activated sludge studies using similar methods also generally reported higher hydrolysis rates than those measured in granules, although hydrolysis rates differed between studies and plants up to two orders of magnitude (e.g. protease activities reported in AS range from 0.015 to 2.1 µmol product mg VSS^−1^ h^−1^) (Frølund et al., 1995; Nybroe et al., 1992). In this study too, the available surface area determined the activity of the sludge, and the hydrolysis rates increased upon granule crushing (data not shown). The starch and proteins used in the assays were soluble substrates: the molecular weight of the labelled starch was lower than 1 kDa; and casein has a molecular weight of around 24 kDa. Considering that the hydrolysis rate of even the small starch molecules was determined by the available granule surface area, the same could be expected for larger substrates.

### 3.2 Hydrolysis sites and hydrolytic capacity distribution within granules

#### 3.2.1 Mass-transfer limited hydrolysis of polymeric substrates occurs at the granule surface

**Fig. 2:**
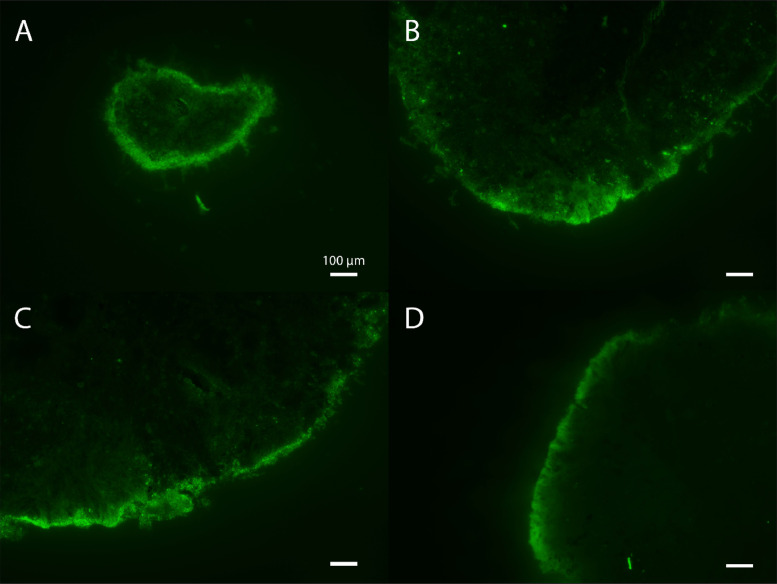
**Fluorescence microscope images of sections of intact granules incubated with BODIPY-casein.** (a) 0.5−1 mm granule; (b) 1.6−2 mm granule; (c) 2.5−3.15 mm granule; (d) 4−4.8 mm granule. All size-bars indicate 100 µm. The areas where BODIPY-casein is hydrolysed appear bright green.

The sites of hydrolysis were microscopically visualized in granules incubated with fluorescent substrates; intact granules were incubated for 2 h and cryosectioned afterwards. Fig. 2 shows the micrographs of granules of 4 different sizes incubated with BODIPY-casein. Amylase assays rendered similar results which can be found in the Supplementary information, as well as additional protease images (Figs. S3−S5). All the granule sizes revealed a hydrolytically active layer in the outer part of the granules. The thickness of this layer varied depending on the zone of the granule, and rarely reached further than the outer 100 µm of granule. Granule size did not appear to impact the thickness of the hydrolytically active layer.

Protease active sites were generally arranged in a thin layer close to the surface of the granules, within the outer 100 µm. In some spots along the granule surface, larger fluorescent patches could be observed which exceeded the 100 µm of depth. Those areas were generally more fluffy (Supplementary information; Fig. S4.2). With protease staining, it was seldom possible to distinguish individual stained cells, but rather a cloudy background was observed, as if hydrolysis products were distributed throughout the extracellular space. For amylase, on the contrary, distinct cells were discernible, even though a fluorescent background was observed too (Supplementary information; Fig. S5). Nevertheless, the granule depth at which hydrolysis occurred was similar for both substrates.

#### 3.2.2 Hydrolytic potential is also present deeper inside the granule

**Fig. 3:**
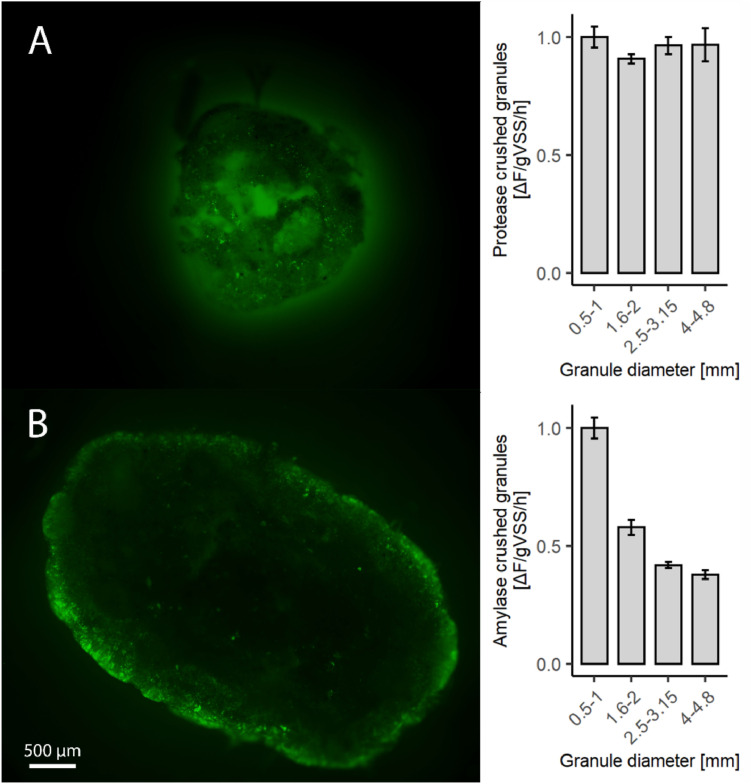
**Micrographs of granules incubated with labelled substrate after sectioning, showing the incubated sectional plane.** Top = granule incubated with Bodipy-casein; bottom = granule incubated with Bodipy-starch. The hydrolytically active granule areas appear bright, due to the fluorescence emitted by hydrolysed BODIPY. On the right, the hydrolytic activity of the crushed granules of different sizes is plotted; each bar represents the average of 4 samples (error bars = standard deviation).

The distribution of hydrolytic potential in granules was further inspected by eliminating mass transfer limitation, that is, slicing granules before incubating them with fluorescent substrates in order to expose the full sectional plane to the fluorescent substrate. The result of these experiments differed for both substrates: while proteases seemed to be distributed throughout the whole granule, amylase activity was still restricted to the outer parts of the granules, although in a thicker layer than when incubating intact granules (Fig. 3).

The activity distribution observed under the microscope related to the hydrolysis rates measured in crushed granules (Fig. 3). When the granule structure was disrupted by crushing, all the granule sizes had the same protease activity, suggesting that the inner parts of the granules had similar hydrolytic capacities across granule sizes. On the contrary, the amylase activity of crushed granules decreased with granule size. Based on the microscopic observations, this could be due to low or no amylase activity in the inner volume of granules, and the larger proportion of such volume in larger granules*.*

The difference in protease and amylase enzyme distribution might be related to the distribution of different macromolecules in the granule matrix. Previous studies investigating the distribution of extracellular polymeric substances (EPS) in lab-grown AGS reported that α-polysaccharides were mainly present close to the granule surface (Adav et al., 2008; McSwain et al., 2005). Proteins, on the other hand, were distributed throughout the granule, and the granule core was mainly composed of protein. The distribution of proteins and polysaccharides in full-scale granules was not studied yet, but might be similar to the reported observations in lab-grown granules. The different distribution of proteases and amylases throughout the granules might reflect the distribution of their potential substrates, proteins and alpha polysaccharides, on the granule matrix. In spite of these differences in granule-wide distribution of protease and amylase activity, the hydrolysis of both casein and starch in intact granules was restricted to the granule surface, indicating that hydrolysis is mass-transfer limited.

#### 3.2.3 Microorganisms in the vicinity of the hydrolytically active layer

**Fig. 4:**
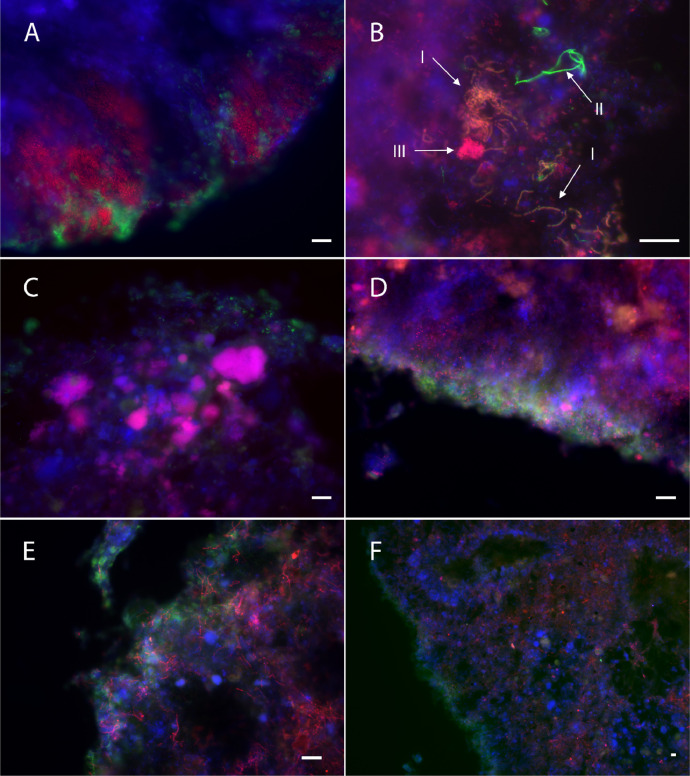
**Fluorescence microscope images of aerobic granules stained with fluorescent substrates followed by FISH.** Blue = EUB; Green = activity staining (amylase in a-c; protease in d-f); Red = specific microbial groups: (a) PAOmix; (b) Actino-221 (*Tetrasphaera*); c) GAOmix; d) Bac111 (*Saprospiraceae*); e-f) CFX1223 + GNSB-941 (*Chloroflexi*), at two different magnifications to visualize granule surface and granule-wide distribution. Size-bar = 20 µm. The arrows in b indicate individual stained cells (I: starch hydrolysing *Tetrasphaera*; II: non-*Tetrasphaera* starch hydrolyser; III: non-starch hydrolysing *Tetrasphaera*).

Microorganisms surrounding the hydrolytically active layer were identified by FISH. PAO, GAO, and putative starch and protein hydrolysers were targeted via FISH probes. GAO and *Ca. Accumulibacter*-related PAO were abundant in all granule sizes. PAO were most concentrated in the outer granule layers, while GAO were abundant in the core too. These microorganisms were often intercalated with hydrolytically active cells (Fig. 4a).

**Fig. 5:**
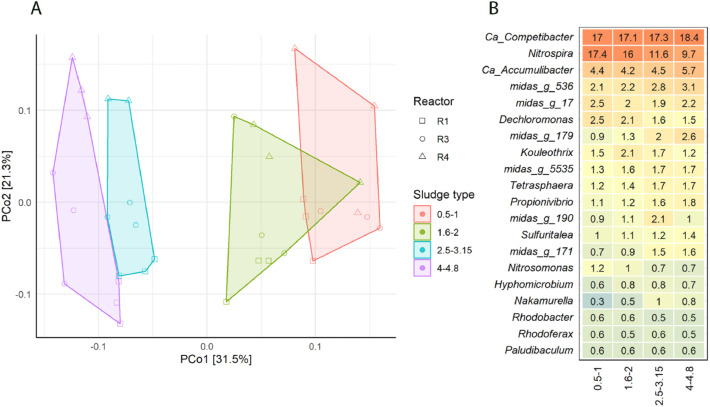
**Microbial differences between aerobic granules of different sizes**. (A) PCoA plot of the Bray-Curtis dissimilarity between samples. Each point represents one DNA sample. (B) Heatmap showing the most abundant genera identified by 16S rRNA amplicon sequencing. The values in the heatmap signify percent of reads of an OTU in three reactors and triplicate samples. All samples were taken in the same day.

The amylase-staining was still visible after the FISH hybridization procedure, making it possible to identify some of the starch hydrolysing organisms. Some of the starch hydrolysers hybridized with the probe Actino-221 (targeting *Tetrasphaera*), specifically some of the filamentous starch hydrolysers (Fig. 5b, arrows I). Some of the *Tetrasphaera* members can hydrolyse starch, and they are overall regarded as dominant glucose fermenters in EBPR plants (Nielsen et al., 2012; Xia et al., 2008a). Despite some of the amylase stained cells were identified as *Tetrasphaera*, most of the starch hydrolysing organisms remained unidentified. In general, the abundance of *Tetrasphaera* was high, as in previous AS and AGS studies (Ali et al., 2019; Nguyen et al., 2011; Stokholm-Bjerregaard et al., 2017; Toja Ortega et al., 2021b).

Since individual cells were seldom stained with protease substrates, protein hydrolysing organisms could not be identified. Nevertheless, it was possible to observe the microorganisms in the vicinity of the active protease sites. Phylum *Chloroflexi* and family *Saprospiraceae* were targeted as putative hydrolysers (Xia et al., 2007). The probes targeting these taxa hybridized with a significant amount of organisms in the sludge. Some of the *Chloroflexi* or *Saprospiraceae* hybridized cells were located close to the hydrolysis sites; however, their distribution was not restricted to those sites, and they were most abundant further from the granule surface. Therefore, their relevance for protein hydrolysis could not be determined. *Saprospiraceae* were rod-shaped, often arranged in colonies, and located mostly in the outer part of the granules. *Chloroflexi* were filamentous and widely distributed throughout the granules. *Chloroflexi*-hybridized cells were specially abundant in the granule core, where they might be subsisting on cell decay products and other recalcitrant compounds (Fig. 4f) (Kragelund et al., 2007). Interestingly, the *Chloroflexi* genus *Kouleothrix* was identified by 16S as one of the abundant genera (Fig. 5b). This genus is considered a major indicator of bulking in AS (Nittami et al., 2017). However, it did not seem to have detrimental effects on aerobic granules. *Chloroflexi* filaments did not result in filamentous outgrowths or irregular granule surfaces (Supplementary information, Fig. S6). They rather constituted a filamentous network inside the granules (Fig. 4e, f). A similar observation was made by De Graaf et al. (2020), who cultivated aerobic granules with a high abundance of the filamentous *Thiothrix*. The high proportion of filamentous organisms in the granules did not deteriorate granule morphology or cause operation issues, showing that operating conditions rather than bacterial morphology determine granule stability (de Graaff et al., 2020). This interpretation is supported by our observation of filamentous organisms thoroughly distributed in stable full-scale granules.

### 3.3 Additional comparison of the different granule size fractions

The microbial composition of granules of different sizes was compared, based on 16S rRNA amplicon sequencing results. Fig. 5b shows the average abundance of the main 20 genera. The abundances of the main microorganisms showed some variation between reactors; for the composition per reactor, check Supplementary information (Fig. S7). Nevertheless, the main genera were shared between all samples. The microbial community composition differed significantly between the different granule size fractions. When studying the Bray-Curtis dissimilarity between samples, one can appreciate that samples cluster according to granule size (Fig. 5a). The distance between samples grows with increasing granule size difference.

In all granule sizes, the microorganisms targeted by FISH were found in high relative abundance in NGS results. However, the differences in relative abundance between granule sizes were small. An increase in *Ca. Competibacter* and *Ca. Accumulibacter* can be noticed with increasing granule size, but the relative abundance differences are not significant (*p* < 0.05/number of comparisons). The relative abundance of the family *Saprospiraceae* did significantly increase from the largest to smallest granule sizes. Other genera that were at significantly higher concentrations in the smaller granules were the nitrifying *Nitrosomonas* and *Nitrospira*, as described previously (Nguyen Quoc et al., 2021). To check other significant abundance differences on the most abundant microorganisms, see Supplementary information (Tables S8.1 and S8.2).

Activity batch tests revealed an increasing specific anaerobic acetate uptake and P release with increasing granule size (Table 1). The terminal settling velocity was also higher in the largest granules, as expected due to their larger size (Liu et al., 2008; van Dijk et al., 2020) (Table 1). These results are in agreement with the observations at Garmerwolde WWTP and the hypothesis that the higher settling velocity of larger granules favours PAO enrichment (Ali et al., 2019; van Dijk et al., 2022). Granule density decreased with increasing granule size, which could be explained by a reduced substrate availability at the granule core due to diffusion limitation leading to biomass decay. The large granules (>4 mm), older and with a larger core volume, were the least dense, in line with results reported by Toh et al. (2003).

**Table 1:** Granule characterization results. Acetate uptake rate, P release rate and granule density were measured in triplicate. Terminal settling velocity was measured for 49-55 granules of each size range. Average values ± standard deviation are shown.

	Acetate uptake rate [mg AcH/g VSS/h]	P release rate [mg P/g VSS/h]	Terminal settling velocity [m/h]	Granule density [g VSS/L]
4-4.8 mm	33 ± 5	10.9 ± 0.8	112 ± 11	55 ± 8
2.5-3.15 mm	23 ± 2	8.9 ± 1.0	86 ± 10	60 ± 4
1.6-2 mm	25 ± 2	8.4 ± 1.2	56 ± 12	71 ± 5
0.5-1 mm	18 ± 1	5.9 ± 0.6	29 ± 11	76 ± 10

### 3.4 General discussion

Our results showed a similar distribution of hydrolytic activity in all the granules ranging from 0.5 to 4.8 mm from a full-scale AGS reactor. All the granules had an active hydrolytic layer at their outer part and its thickness did not seem to depend on granule size. The hydrolysis rates measured in the different sized granules also indicated that hydrolysis is largely surface-area related. This makes small granules more suitable to degrade polymeric substrates; not because of a specific microbial community composition or higher enzyme concentration, but mainly due to their high available surface area. Their activity was enhanced by a somewhat higher surface-related activity, although a significant trend could not be identified.

There was, nonetheless, microbial differentiation between granules of different sizes. The microbial community of the granules shifted with the changes in granule size. Since most hydrolysers remained unidentified, it was not possible to assess their abundance changes in the different size fractions. The abundances of the PAO *Ca. Accumulibacter* and the GAO *Ca. Competibacter* did not differ significantly between granule size fractions. Nonetheless, activity tests did show differences in acetate uptake and P release rates. The largest granules exhibited higher acetate uptake and P release activities, reflecting a higher EBPR activity. This higher activity can be explained by the differences in substrate availability during full-scale operation. Due to stratification during settling, larger granules will be located closer to the influent inlet at the bottom of the reactor than the small granules (van Dijk et al., 2020, van Dijk et al., 2022). Large granules thus spend most of the anaerobic period in contact with the influent, while smaller granules are only in contact with the influent during the final stages of the feeding phase, when the influent level reaches the upper part of the settled bed (van Dijk et al., 2022). VFAs are taken up at high rates, and hence, the position of granules in the sludge bed during feeding impacts their access to VFA significantly (de Kreuk and van Loosdrecht, 2004; Pronk et al., 2015a). In this way, anaerobic COD storage is promoted in granules at the bottom of the settled granule bed, that is, in the largest granules (van Dijk et al., 2022). This may lead to PAO and GAO enrichment as previously reported by Ali et al. (2019) in the full-scale AGS from WWTP Garmerwolde.

In contrast, hydrolysis potential was similar, yet surface-bound, across granule sizes. One reason could be that the position of the granules in the settled bed has little effect on their interaction with polymeric substrates. Granule-substrate interaction is greatly influenced by the size of the substrate and by mixing. The majority of polymeric substrates in domestic wastewater are in the particulate form. Polymeric substrates can also be soluble and colloidal, but these forms are generally less abundant in domestic wastewater; likely due to biodegradation during transport in the sewer. Reported soluble protein and carbohydrate concentrations in domestic wastewater range between 3 and 100 mg COD/L, and between 5 and 110 mg COD/L, respectively. Their particulate counterparts range between 3 and 161 mg COD/L for proteins and between 5 and 200 mg COD/L for carbohydrates (Narkis et al., 1980; Raunkjær et al., 1994; Ravndal et al., 2018; Toja Ortega et al., 2021a, 2021b). During plug-flow feeding, while soluble and colloidal substrates penetrate the granules easily, particulates are trapped within the voids of the granule bed, but their attachment to the biomass appears to be low (Layer et al., 2020; Ranzinger et al., 2020). Most of the particulate COD is thus likely resuspended in the aerobic phase, where the reactor volume is fully mixed. Aeration creates a turbulent flow which promotes collision between granules and particulate substrates (Boltz and La Motta, 2006; Layer et al., 2020); Layer et al. (2020) reported a retention of 60-85% of the particulate COD in AGS after 3 h of aeration. Hence, the aerobic phase probably determines the biomass fraction particulates can attach to, including both the granular and flocculent fractions that usually constitute the AGS bed. In this context, the development of hydrolytic capacity would be driven by the interactions taking place during the aerobic phase, where there is no sludge stratification, and a large surface area is advantageous to enhance collision chances.

Furthermore, the synthesis of hydrolytic enzymes within the granule matrix might be triggered by compounds in the EPS rather than by substrates in the bulk liquid. Studies on the EPS composition of AGS found proteins widespread through the granules, and α-polysaccharides only in the outer layers (Adav et al., 2008; McSwain et al., 2005). The granule-wide distribution of hydrolytic activity determined in our study also showed protease activity all through the granule, while amylase was restricted to the surface. High protease activities have been measured previously in the granule core, and related to EPS turnover and biomass decay (Adav et al., 2009). Therefore the hydrolytic enzymes could be mainly degrading EPS and cell decay components, although they also allow granules to hydrolyse polymeric substrates from the influent, at the surface of the AGS or within the granules in case of diffusible polymers. The combined FISH and activity staining results showed that PAO and GAO localized close to the hydrolysis sites, and they could potentially take up the hydrolysates generated during the anaerobic phase. Furthermore, some of the actinobacterial PAO (*Tetrasphaera*) in this study could hydrolyse starch, also suggesting that polysaccharides can be used for EBPR. However, the contribution of polymeric COD to EBPR remains a topic for further research.

Evident from our study is that granule surface area will determine the potential of aerobic granules to degrade polymeric COD, both aerobically and anaerobically. If boosting the anaerobic uptake of polymeric COD is desired, due to a wastewater with low VFA for example, a smaller average granule size might be beneficial. The length of the anaerobic phase could be regulated too to promote hydrolysis. Smaller granules will also increase nitrification rates, and overall improve mass-transfer (Nguyen Quoc et al., 2021). If, on a different scenario, there are enough VFA in the influent for effective EBPR, larger granules might be preferred for an improved simultaneous nitrification-denitrification (SND) and a decreased degradation of polymeric substrates. Lower aerobic COD degradation would then decrease aeration costs and enhance the recovery of energy from the sludge spill (Guo et al., 2020).

## 4 Conclusions

The main conclusions of this study are:

1) The stratification of the AGS granule bed during feeding drives microbial differentiation in granules of different sizes, based on their proximity to the influent inlet during feeding. As a result, large granules have higher acetate uptake rates, reflecting their preferential access to influent VFA. Nevertheless, this differentiation is not evident from the distribution of hydrolysis capacity. All granule sizes exhibited a similar hydrolytic activity per surface area, and thus the development of hydrolytic activity does not appear to be conditioned by the position of granules in the settled bed during feeding.
2) Hydrolysis of polymers occurred in the outer 50−100 µm of the granules. PAOs and GAOs were observed in the vicinity of the hydrolytically active granule sites, but their anaerobic uptake of hydrolysates was not demonstrated and deserves further research.
3) Small granules returned higher specific hydrolysis rates due to their larger specific surface area. Thus, small granules are preferred when anaerobic COD storage from polymeric substrates needs to be maximized. However, the hydrolysis kinetics of polymeric substrates should be studied further to evaluate how significantly these substrates can contribute to anaerobic COD storage in AGS.

## Declaration of Competing Interest

The authors declare that they have no known competing financial interests or personal relationships that could have appeared to influence the work reported in this paper.
